# Recombinant human soluble thrombomodulin administration improves sepsis-induced disseminated intravascular coagulation and mortality: a retrospective cohort study

**DOI:** 10.1186/1477-9560-11-3

**Published:** 2013-02-18

**Authors:** Takahiro Kato, Takamasa Sakai, Miki Kato, Mao Hagihara, Takaaki Hasegawa, Katsuhiko Matsuura, Takashi Nakagawa

**Affiliations:** 1 Department of Pharmacy, Aichi Medical University, 1-1 Yazakokarimata, Nagakute, Aichi 480-1195, Japan; 2 Laboratory of Drug Information, Meijo University Faculty of Pharmacy, 150 Yagotoyama, Tempaku-ku, Nagoya, Aichi 468-0077, Japan; 3 Department of Infection Control and Prevention, Aichi Medical University, 1-1 Yazakokarimata, Nagakute, Aichi 480-1195, Japan; 4 Department of Emergency and Critical Care Medicine, Aichi Medical University, 1-1 Yazakokarimata, Nagakute, Aichi 480-1195, Japan

**Keywords:** Disseminated intravascular coagulation, Sepsis, Thrombomodulin, Intensive care unit, Critically ill patient, Anticoagulant, Multiple organ failure, DIC score, JAAM

## Abstract

**Background:**

Early treatment of disseminated intravascular coagulation (DIC) can be associated with improved patient outcomes. The Japanese Ministry of Health and Welfare (JMHW) and the International Society on Thrombosis and Haemostasis (ISTH) criteria are the most specific for diagnosis of septic DIC. The revised Japanese Association for Acute Medicine (JAAM) criteria are able to diagnose sepsis-induced DIC in the early stage. Recombinant human soluble thrombomodulin (rhTM) has recently been used for treating DIC. Previous studies have shown a benefit of using rhTM for D,IC diagnosed by the JMHW or ISTH criteria, but not the JAAM criteria. The purpose of this study was to sequentially evaluate coagulation biomarkers and the DIC score after giving rhTM treatment to patients with sepsis-induced DIC diagnosed according to the JAAM criteria.

**Methods:**

We performed a retrospective cohort study. Critically ill patients were included if diagnosed with sepsis-induced DIC according to the JAAM criteria. They were either treated without rhTM (control group) or with rhTM (treatment group). The primary outcome was the DIC score on day 7. The secondary outcome was 28-day mortality from the start of DIC treatment. Changes in the results of coagulation tests were assessed over time from the start of treatment to day 7.

**Results:**

Twelve and 23 patients were assigned to the treatment and control groups, respectively. The DIC score on day 7 was significantly higher in the treatment group (3.3 ± 1.4) than in the control group (4.9 ± 1.8, *p* < 0.05). Estimated survival showed lower in treatment group than control group. There was significant difference between the control group and the treatment group (*p* < 0.05). The D-dimer level on day 7 was significantly lower in the treatment group (7.5 ± 4.1 μg/mL) than in the control group (30.9 ± 33.6 μg/mL, *p* < 0.05). Life-threatening bleeding did not occur. Our results indicated that rhTM improved sepsis-induced DIC and mortality.

**Conclusions:**

Recombinant human soluble thrombomodulin may improve sepsis-induced DIC diagnosed according to the JAAM criteria without an increased bleeding risk.

## Background

Disseminated intravascular coagulation (DIC) is associated with high mortality in patients with severe sepsis. Excessive coagulation activation, inhibition of fibrinolysis, and consumption of coagulation inhibitors lead to a hypercoagulable state, resulting in fibrin deposition in microvessels and inflammatory reactions. Although the effectiveness of anticoagulant therapy in septic patients is still controversial worldwide, some studies have suggested that rapid diagnosis and early treatment of DIC improve outcomes for these patients. In particular, therapeutic intervention directly against coagulation and inflammation in DIC associated with severe sepsis is effective [1,2], and it is generally accepted that early, aggressive treatment of the underlying disease is important.

Recently, the International Society on Thrombosis and Haemostasis (ISTH) criteria were proposed by the subcommittee of the ISTH. The revised Japanese Association for Acute Medicine (JAAM) criteria were proposed by the JAAM, and the Japanese Ministry of Health and Welfare (JMHW) criteria were proposed by the JMHW. These criteria have been used for clinical diagnosis of DIC [3-5]. The JAAM criteria have acceptable validity for diagnosis of DIC in the early phase of disease, and the scoring system can diagnose DIC with a higher sensitivity than can the criteria of the ISTH for overt DIC [6], thus enabling patients to receive early treatment.

The novel biological agent recombinant human soluble thrombomodulin (rhTM) was recently approved and has been used clinically for DIC treatment in Japan. The clinical effects of rhTM on DIC were previously examined in patients diagnosed according to the JMHW criteria in a multicenter randomized clinical trial conducted in Japan [7]. This study revealed significantly better resolution of DIC in the rhTM treatment group than in the heparin treatment group. Only non-significant trends in favor of rhTM compared with heparin were observed for mortality in patients with sepsis-induced DIC [7]. Yamakawa *et al.* showed that administration of rhTM reduced 28-day mortality in patients with severe sepsis [8].

However, the clinical effects of rhTM on patients diagnosed in the early stage of sepsis-induced DIC according to the JAAM criteria have not been assessed. Because the JAAM criteria show higher sensitivity for DIC diagnosis than do the ISTH criteria, and because early treatment of DIC may be associated with improved outcomes [1], rhTM treatment in patients diagnosed with sepsis-induced DIC according to the JAAM criteria may enhance clinical outcomes. Therefore, the purpose of this study was to sequentially evaluate coagulation biomarkers and the DIC score in rhTM treatment of patients with sepsis-induced DIC diagnosed according to the JAAM criteria.

## Methods

### Patients and study design

**Figure 1:**
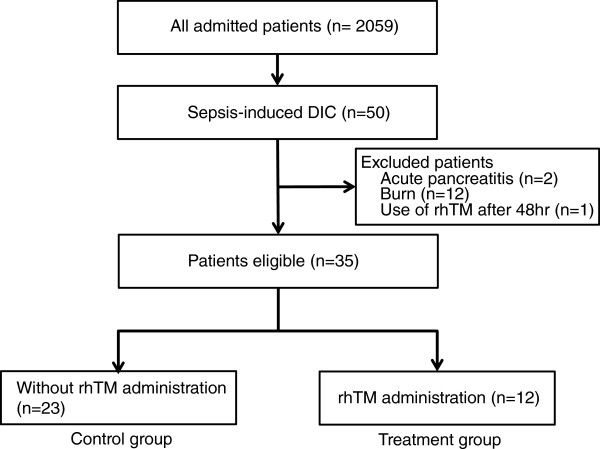
**Patient flow diagram.** DIC, disseminated intravascular coagulation; rhTM, recombinant human soluble thrombomodulin.

A retrospective analysis of all patients admitted to the intensive care unit (ICU) of Aichi Medical University Hospital between May 2008 and March 2011 was performed. Although the criteria for ICU admission were not standardized, all patients included in this study were diagnosed with sepsis-induced DIC according to the JAAM criteria. Administration of rhTM started within 48 h from the initiation of DIC treatment. Treatment with rhTM (0.06 or 0.02 mg/kg/day; patients who required renal replacement therapy for acute kidney injury [AKI] were drip-infused for 30 min once daily) was continued for 7 days. There was no difference in treatment strategy among all patients, equipment used, or number of physicians and nurses who took care of the patients during the study period. All patients were principally treated according to the strategy of the Surviving Sepsis Campaign Guidelines [9]. Any patients who lacked the required laboratory data were excluded, and other exclusion criteria were as follows: acute pancreatitis, burns, treatment with danaparoid sodium, fatal or life-threatening bleeding (defined as intracranial or pulmonary bleeding) at the start of treatment for DIC, and age of ≤15 years. A patient flow diagram is shown in Figure 1.

The protocol was reviewed and approved by a local institutional review board. Informed consent was not required because blood samples were taken as part of the routine patient care for clinical laboratory testing, but the highest standard of privacy policy was applied.

### Data collection

Relevant clinical background, medication history, and laboratory data of all patients were collected at appropriate times during the treatment for sepsis. The variables compared between the two groups were age, sex, Acute Physiologic and Chronic Health Evaluation II (APACHE II) score, Sequential Organ Failure Assessment (SOFA) score, number of dysfunctional organs, shock (hypotension not reversed with fluid resuscitation), presence of AKI as defined by the Acute Kidney Injury Network [10], acute respiratory distress syndrome (ARDS), DIC score (calculated with the JAAM criteria) at the start of DIC treatment, maximum DIC score during DIC treatment, number of patients who fulfilled the ISTH criteria, platelet count, prothrombin time (PT) ratio, D-dimer level, antithrombin III (AT III) activity, renal replacement therapy, mechanical ventilation, use of vasopressors, administration of AT III, low-dose steroids, platelet concentrate, fresh frozen plasma transfusion, rate of positive blood culture, and site of infection. Patients were followed until day 28 after the start of DIC treatment.

### Evaluation of clinical response

The DIC score and DIC resolution rate (rate of recovery from DIC) were assessed 7 days after the start of DIC treatment (or withdrawal) based on the JAAM criteria as the primary efficacy endpoint. Secondary endpoints were as follows: the 28-day mortality from the start of DIC treatment; severity of the patients’ conditions as calculated by the SOFA score from the start of treatment to day 28; and changes over time in the results of coagulation tests, such as the platelet count, D-dimer level, PT ratio, fibrinogen level, and AT III activity, were also assessed from the start of treatment to day 7. In addition, the presence of serious adverse events related to bleeding (defined as intracranial hemorrhage, gastrointestinal or respiratory tract bleeding that was uncontrollable by conservative treatments, and bleeding at a critical location, such as retinal hemorrhage, major hemarthrosis, spinal hemorrhage or any life-threatening bleeding) that led to discontinuation of the administrated study drug and abnormal changes in clinical laboratory test results related to the diagnosis of DIC were investigated.

### Statistical analysis

Data are expressed as group means ± standard deviation or percentages, as appropriate. Continuous variables were compared between groups using the Student’s *t*-test. Noncontinuous variables were compared between groups using the Mann–Whitney test. Categorical variables were analyzed using the *χ*^2^ test or Fisher’s exact test, as appropriate. Univariate analysis of the time to DIC resolution and the mortality rate were compared using Wilcoxon’s test. In addition, stepwise multivariate Cox regression analysis was used to assess the covariates that were associated with time to mortality. Adjusted curves of time to mortality by associated covariates were estimated. Comparisons of DIC scores, SOFA scores, D-dimer level, platelet count, PT ratio, fibrinogen, and AT III activity between groups over time were analyzed by repeated measures analysis of variance (ANOVA) adjusted for the baseline values as a covariate, and by *post hoc* Bonferroni test. The last-observation-carried-forward method for missing data was used for the analysis. Missing samples occurred because some samples were not drawn. A *p* value of <0.05 was considered statistically significant. Statistical analyses, except for ANOVA and the *post-hoc* Bonferroni test, were performed using JMP for Windows version 5.0.1 software (SAS Institute, Inc, U.S.). ANOVAs and *post hoc* Bonferroni tests were performed using Microsoft Excel 2010 (Microsoft Corporation, U.S.).

## Results

### Baseline characteristics

During the study period, although 49 patients were diagnosed with DIC, only 35 patients met the requirements of our study. Fourteen patients were excluded (acute pancreatitis, 2; burns, 12). Twelve patients were treated with rhTM for DIC (treatment group) and 23 patients were treated without rhTM (control group). All patients were regarded as having been infected with pathogens. Baseline characteristics and therapeutic interventions of the study population are shown in Table 1. Twenty-four patients were male. The duration of rhTM administration was 3 to 7 days (5.83 ± 1.26 days). Baseline characteristics, including severity of underlying disease, DIC scores, and treatment excluding rhTM administration showed no significant differences between the control and treatment groups. The ratio of patients diagnosed with DIC according to the ISTH criteria was 39.1% (9 of 23) for the control group and 41.7% (5 of 12) for the treatment group.

**Table 1:** Characteristics and diagnostic data of the patients

	**Control group (n = 23)**	**Treatment group (n = 12)**	***P *value**
Age	70.9 ± 13.7	60.6 ± 19.2	0.117
Male (%)	16 (69.6)	8 (66.7)	0.919
APACHE II score	29.2 ± 8.5	26.6 ± 8.6	0.395
SOFA score (day 1)	11.7 ± 3.4	10.5 ± 2.5	0.418
Number of dysfunctional organs	3.7 ± 1.0	3.5 ± 0.7	0.450
Shock	11 (47.8)	5 (41.7)	0.728
AKI	13 (56.5)	5 (41.7)	0.404
ARDS	15 (65.2)	8 (66.7)	0.618
DIC score (day 1)	5.8 ± 1.2	5.7 ± 1.4	0.881
DIC score (max)	6.7 ± 1.02	6.33 ± 1.33	0.703
ISTH criteria (%)	9 (39.1)	5 (41.7)	0.884
Platelet count (10^3^/μl)	94.3 ± 49.6	65.6 ± 48.3	0.130
Prothrombin time ratio	1.52 ± 0.39	1.48 ± 0.41	0.759
D-dimer (mg/ml)	26.9 ± 35.3	55.1 ± 133.1	0.486
AT III activity (%)	51.3 ± 19.0	61.3 ± 20.5	0.264
Renal replacement therapy (%)	13 (56.5)	8 (66.7)	0.559
Mechanical ventilation (%)	23 (100)	12 (100)	-
Use of vasopressor (%) ^†^	21 (91.3)	10 (83.3)	0.482
Use of low-dose steroid (%)	1 (4.35)	1 (8.33)	0.630
Use of AT III (%)	16 (69.6)	5 (41.7)	0.111
Use of Platelet concentrate (%)	10 (43.5)	8 (66.7)	0.189
Use of fresh frozen plasma (%)	15 (65.2)	8 (66.7)	0.932
Positive blood culture (%)	7 (31.8)^*^	7 (58.3)	0.134
Period for rhTM administration (day)	-	5.8 ± 1.3	-
Sites of infection			
Lung (%)	9 (39.1)	4 (33.3)	0.735
Abdomen (%)	7 (30.4)	4 (33.3)	0.735
Urinary tract (%)	2 (8.7)	2 (16.7)	0.364
Other (%)	5 (21.8)	3 (25.0)	0.203

No significant differences were observed between the control and treatment groups.

*Blood culture was not performed in one patient.

^†^Vasopressors not only were used for sepsis but also before the onset of sepsis-induced DIC and were used to maintain circulation for surgery.

### Primary endpoint

**Figure 2:**
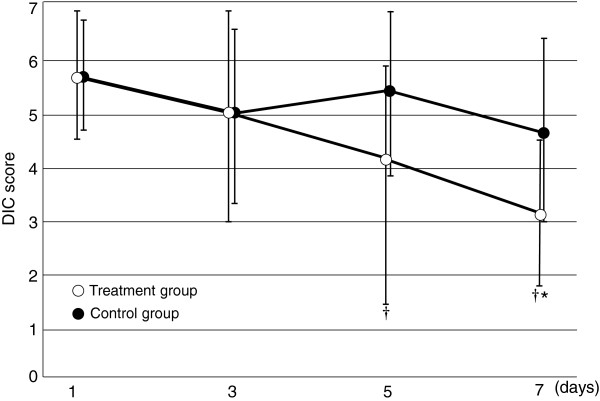
**Serial changes in the DIC score in the two groups.** Data are expressed as group means ± standard deviation. The DIC score decreased over time in the treatment group (*p* < 0.05). The interaction between treatment and time was statistically significant (p < 0.01). The degree of decrease in the DIC score was significantly greater in the treatment group than in the control group. *p < 0.05 compared with the control group, ^†^*p* < 0.05 compared with baseline.

**Figure 3:**
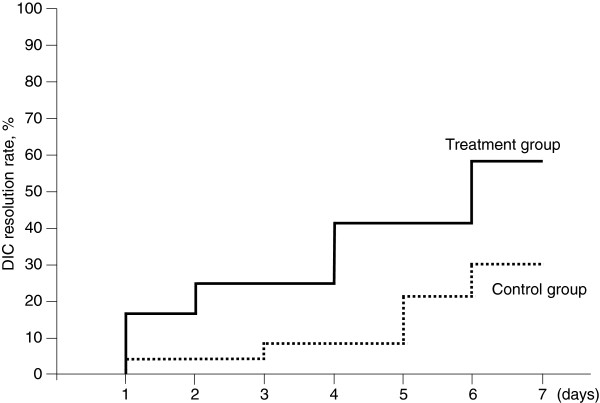
**DIC resolution curves in Wilcoxon models.** The solid line represents patients in the treatment group, and the dotted line represents patients in the control group. The DIC resolution rate increased over time in both groups. The DIC resolution rate was improved to a greater degree in the treatment group than in the control group (*p* = 0.079).

On day 7, the DIC score was significantly lower in the treatment group (3.3 ± 1.4) than in the control group (4.9 ± 1.8, *p* < 0.05, Figure 2). The DIC resolution rate was significantly higher in the treatment group (58.3%) than in the control group on day 7 (26.1% *p* = 0.079, Figure 3). While three patients in the control group died before 7 days, no patient in the treatment group died before 7 days.

### Secondary endpoints

**Figure 4:**
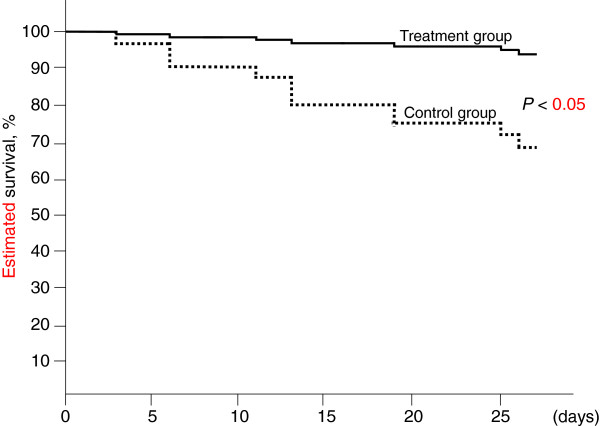
**Adjusted estimated survival curves by the covariate of AKI in Cox regression models.** The solid line represents patients in the treatment group, and the dotted line represents patients in the control group. Treatment with rhTM was associated with a significantly higher rate of survival (P = 0.026 by Cox regression analysis). AKI, acute kidney injury; rhTM, recombinant human soluble thrombomodulin.

**Figure 5:**
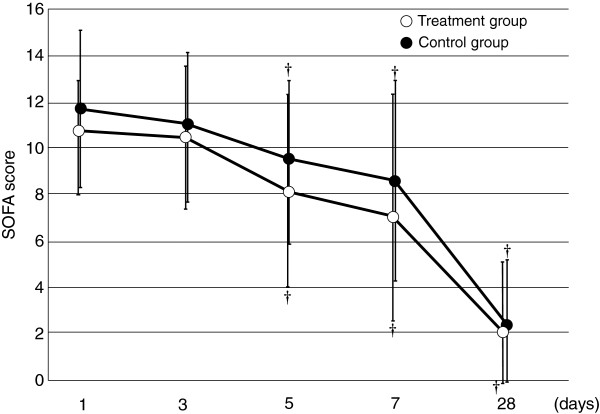
**Serial changes in the SOFA score in the two groups.** Data are expressed as group means ± standard deviation. Although the SOFA score was not significantly different between the two groups (*p* = 0.493), the interaction between treatment and time was statistically significant (p < 0.001). ^†^*p* < 0.05 compared with baseline.

**Figure 6:**
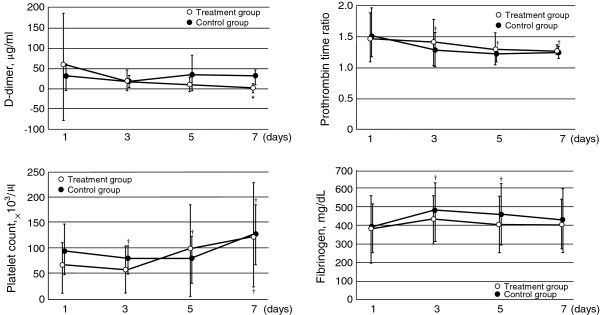
**Serial changes in coagulation tests in the two groups.** Data are expressed as group means ± standard deviation. There were no interaction between treatment and time in D-dimer (*p* = 0.289), Prothrombin time ratio (*p* = 0.457) and Fibrinogen level (*p* = 0.528). *p < 0.01 compared with the control group. ^†^*p* < 0.05 compared with baseline.

**Figure 7:**
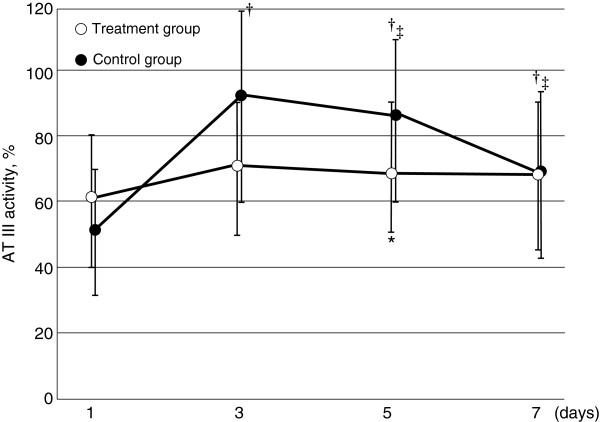
**Serial changes in AT III activity in the two groups.** Data are expressed as group means ± standard deviation. There were no interaction between treatment and time (*p* = 0.618). Changes over time of AT III activity were not significantly different in the treatment group, and they showed a significant decrease compared with the maximum level and day 5 and 7 in the control group. *p < 0.01 compared with the control group, ^†^*p* < 0.05 compared with baseline, ^‡^*p* < 0.05 compared with day 3.

The 28-day mortality rate in the control group was 33.3% (8 of 23 patients) compared with 8.3% (1 of 12 patients) in the treatment group (*p* = 0.075 by Wilcoxon’s test). We performed Cox regression analysis to adjust for the baseline variables of patients. We assessed a total of 11 possible confounders related to outcome: age, sex, APACHE II score, SOFA score, fulfilled ISTH criteria at the start of DIC treatment, AKI, ARDS, use of AT III, platelet concentrate, fresh frozen plasma, and administration of rhTM. Consequently, two prognostic variables were selected: AKI and administration of rhTM. After adjusting for AKI and rhTM administration was identified as an independent significant predictor of the probability of 28-day mortality (adjusted hazard ratio, 0.089 to 0.904; P = 0.026) (Table 2). The survival curves of the prediction model calculated by Cox regression analysis are shown in Figure 4. Estimated survival showed higher in treatment group than control group. There was significant difference between the control and the treatment groups (*p* < 0.05). The SOFA score was not significantly different between the two groups (Figure 5). In the coagulation tests, D-dimer level was significantly lower on day 7 (7.5 ± 4.1 μg/ml in the treatment group vs 30.9 ± 33.6 μg/ml in the control group; *p* < 0.01), while the platelet count, PT ratio, and fibrinogen levels were not significantly different at day 7 between the groups (Figure 6). AT III was used when the physician considered that it was required. AT III was administrated at a concentration of1500 IU per day for less than 3 days. AT III was not administrated after day 4. AT III activity was not significantly different at day 7 between the two groups, and showed a significant difference compared with the maximum level and day 7 in the control group (Figure 7).

**Table 2:** Independent variables in final multiple regression models by Cox regression analysis

**Variables**	**Coefficient**	**Hazard ratio**	**95% CI**	***P *value**
rhTM administration	−0.958	0.384	0.088 to 0.904	0.026
AKI	0.840	2.316	1.132 to 6.016	0.020

*rhTM*, recombinant human soluble thrombomodulin; *AKI*, acute kidney injury; *CI*, confidence interval.

Until 28 days after the start of DIC treatment, one serious adverse event related to bleeding occurred in the control group (4.3%) and no serious adverse events occurred in the treatment group (0%) within 28 days. No clear correlation was shown between these adverse events and the treatment used. There was no significant difference in the incidence of these adverse events between the two groups (*p* = 1.00).

## Discussion

The results of this study provide evidence that rhTM may have a beneficial effect on coagulation in patients with sepsis-induced DIC diagnosed according to the JAAM criteria. We showed a significant decrease in the DIC score in the treatment group compared with that in the control group. The DIC resolution rate was higher and the mortality rate was lower in the treatment group than in the control group. Bleeding showed no significant difference between the two groups.

Early treatment of DIC may be associated with improved outcomes [1]. In our study, the number of patients who fulfilled the ISTH criteria did not show a significant difference between the two groups. In the present study, approximately half of the patients were in the early phase of DIC compared with the diagnosis according to the ISTH criteria. Mortality has been shown to be correlated with the maximum DIC score [5]. In our study, the maximum DIC score, APACHE II score, SOFA score, AKI, and ARDS showed no significant differences between the control and treatment groups at baseline. Therefore, it was considered reasonable to compare the two groups.

Several DIC markers were assessed. It was difficult to assess the platelet count and AT III activity because they varied widely among the patients, and platelet concentrate was used for approximately half of the patients in the two groups. However, we consider that the contribution of rhTM administration to the increases in platelet counts was not large because there was no significant difference in improvement in the platelet counts between the two groups. In addition, we consider that the contribution of platelet concentrate to the DIC score was not large because the platelet count was <80,000/μl after the use of platelet concentrate at approximately day 7. Although AT III activity decreased from days 3 to 7 in the control group, it did not decrease with rhTM administration. This suggests that rhTM improves sepsis-induced DIC by not decreasing AT III activity. Recombinant human soluble thrombomodulin decreases thrombin-antithrombin complex compared with heparin [7]. Therefore, in the present study, AT III activity did not decrease in the treatment group.

The sensitivity of a low fibrinogen level for the diagnosis of DIC according to ISTH criteria was 28% and hypofibrinogenemia has been detected in severe cases of DIC only [11]. Although rhTM improves fibrinogen levels in animal studies [12,13], fibrinogen levels were normal in all patients in the present study. Therefore, fibrinogen levels showed no significant differences between the control and treatment groups.

D-dimer levels were significantly decreased in the treatment group compared with the control group on day 7. In a phase III trial of rhTM for patients with DIC, Saito *et al.* showed that the rate of change in D-dimer levels in the rhTM group was significantly greater than that in the heparin group, suggesting that rhTM is superior to heparin in the attenuation of the hypercoagulable state [7]. Yamakawa *et al.* showed that rhTM decreased fibrin degradation products in patients with DIC who fulfilled the ISTH criteria [8]. Therefore, it is thought that rhTM affects the treatment of not only overt DIC, but also the early phase of DIC as diagnosed by the JAAM criteria. Recombinant human soluble thrombomodulin directly inhibits thrombin and produces activated protein C (APC), and it induces 50-fold increased inhibition of prothrombinase compared with heparin, thus producing an anti-thrombin effect [12,14]. Although it is considered that regulation of coagulation and activation of fibrinolysis are mechanisms of decreasing D-dimer levels, rhTM does not affect the fibrinolytic system [15]. Therefore, rhTM suppresses the hypercoagulative state in patients with sepsis-induced DIC. As a result, the D-dimer level was decreased and the PT ratio did not decrease in the treatment group in our study.

Although the accuracy of the mortality rate was questionable because of the small sample size, Cox regression analysis indicated that 28-day mortality of the patients treated with rhTM was significantly improved compared with that in the patients treated without rhTM.

In our study, the SOFA score did not show a significant difference on days 7 or 28. Although Yamakawa *et al.*[8] showed that rhTM did not decrease the SOFA score on day 7 compared with the control group, organ damage was improved on days 21 and 28 by rhTM administration, and the 28-day mortality was significantly improved. They investigated patients with DIC diagnosed according to the ISTH criteria. However, in the present study, we investigated patients with DIC diagnosed according to the JAAM criteria. SOFA score might have been nonsignificant because of the small sample size.

Several animal studies have demonstrated a reduction in mortality with the administration of rhTM in severe sepsis models. Iba *et al.*[13] showed that changes in coagulation abnormalities were reduced and that mortality was decreased by the concomitant administration of rhTM and antithrombin in rats with lipopolysaccharide infusion-induced sepsis, which are similar results to our study. Therefore, decreased mortality is expected, not only in patients with overt DIC, but also in patients in the early phase of DIC as diagnosed by the JAAM criteria. D-dimer levels are decreased by recombinant APC [16]; however, APC does not reduce the 28-day mortality [17]. Recombinant human soluble thrombomodulin treatment might have the same effect. Further evaluation of the effects of rhTM is necessary.

Bleeding is the most significant adverse event associated with the administration of rhTM, as it is with recombinant human APC [18]. Sadaka *et al.* reported that life-threatening bleeding was higher in the APC group than in the placebo group [19]. In the present study, the PT ratio and bleeding were not significant between the two groups. Moreover, life-threatening bleeding did not occur. Recombinant human soluble thrombomodulin was considered not to increase the risk of bleeding based on our study results. Clinical doses of APC and rhTM do not affect the clotting time [12,20]. Therefore, PT is not a predictive factor of bleeding by rhTM. This is supported by the fact that there was no significant difference in bleeding between the two groups; the anticoagulative effect of rhTM depends on the amount of thrombin available. Accordingly, after controlling thrombin generation by rhTM administration, rhTM does not work in excess and generation of further APC decreases. Recombinant human soluble thrombomodulin has been shown to have a wider safety margin and a favorable antithrombotic profile with less bleeding than heparin in animals and in *in vitro* experiments [12].

We acknowledge several limitations of our observational study design. This study was not a randomized controlled trial, and we compared two groups retrospectively. A small number of patients was included in this study. Finally, this study was carried out in a single institution. Further multicenter, prospective, randomized trials are necessary to fully evaluate the effects of rhTM.

## Conclusions

Recombinant human soluble thrombomodulin administration may improve sepsis-induced DIC diagnosed according to the JAAM criteria without an increased bleeding risk. Further clinical investigations are necessary to fully evaluate the effect of rhTM.

## Abbreviations

DIC: Disseminated intravascular coagulation; ISTH: Subcommittee of the international society on thrombosis and haemostasis; JAAM: Japanese association for acute medicine; JMHW: Japanese ministry of health and welfare;rhTM: Recombinant human soluble thrombomodulin; ICU: Intensive care unit; AKI: Acute kidney injury; APACHE II: Acute physiologic and chronic health evaluation II; SOFA: Sequential organ failure assessment; ARDS: Acute respiratory distress syndrome; AT III: Antithrombin III; PT: Prothrombin time; ANOVA: Analysis of variance; APC: Activated protein C.

## Competing interests

The authors declare that they have no competing interests.

## Authors’ contributions

TK, TS, MK, MH, TH, KM, and TN contributed to the study conception and design, collected and assembled the study data, contributed to the writing and revising of the manuscript, and provided final approval of the manuscript. TK and TS performed the statistical analysis. All authors read and approved the final manuscript.
